# A Portable Fluorescent Hydrogel-Based Device for On-Site Quantitation of Organophosphorus Pesticides as Low as the Sub-ppb Level

**DOI:** 10.3389/fchem.2022.855281

**Published:** 2022-04-29

**Authors:** Tuhui Wang, Lening Zhang, Hua Xin

**Affiliations:** Department of Thoracic Surgery, China−Japan Union Hospital, Jilin University, Changchun, China

**Keywords:** fluorescent sensor, hydrogel test kit, organophosphorus pesticide, on-site detection, smartphone

## Abstract

Portable devices possess powerful application prospects in on-site sensing without the limitation of bulky instruments. Given the relevance of pesticides to food safety, we herein fabricated a robust gold nanocluster (AuNC)-based hydrogel test kit for precisely quantified chlorpyrifos by using a three-dimensional (3D) printed subsidiary device. In this work, the fluorescence of AuNC-based hydrogel could be efficiently quenched by cobalt oxyhydroxide nanoflakes (CoOOH NFs) through the Förster resonance energy transfer effect. Chlorpyrifos as an acetylcholinesterase inhibitor controls the enzymatic hydrolysis reaction and further regulates the production of thiocholine that could decompose CoOOH nanoflakes into Co^2+^, resulting in the fluorescence response of AuNC-based hydrogel. By using a homemade subsidiary device and smartphone, the fluorescence color was transformed into digital information, achieving the on-site quantitative detection of chlorpyrifos with the limit of detection of 0.59 ng ml^−1^. Owing to specific AuNC signatures and hydrogel encapsulation, the proposed fluorescence hydrogel test kit displayed high sensitivity, good selectivity, and anti-interference capability in a real sample analysis, providing great potential in on-site applications.

## Introduction

Organophosphorus pesticides (OPs) have a crucial role in efficiently protecting modern agriculture from damage by pests (He et al., 2021; Wang T. et al., 2021). Unreasonable usage of OPs will inevitably cause an increase in pesticide residues in agricultural foods, leading to environmental bioaccumulation and further causing huge health hazards to humans (Cui et al., 2021; Dong et al., 2021; Li Q. et al., 2021; Mariyappan et al., 2021; Yao et al., 2022). Hence, there is a stringent demand to exploit simple, effective, and fast-sensing technologies for the analysis of OP residues. Although conventional laboratory-based strategies are accessible for detecting OP residues, containing mass spectrometry, high-performance liquid chromatography, and gas chromatography, their on-site detection capacity is seriously restricted because of the time-consuming procedures and expensive equipment (Fang et al., 2021; Guo et al., 2021). Therefore, exploring new sensors to trace OP residues that are cost-efficient and easy-to-carry is essential for real-time application (Fang et al., 2020; Hua et al., 2021; Li H. et al., 2021; Luo et al., 2021; Li et al., 2022).

In recent years, real-time sensing platforms have been explored for on-site pesticide analysis (Li et al., 2021b; Xu Y. et al., 2021; Farahmand Nejad et al., 2021; Fuentes-Chust et al., 2021). As a typical point-of-care testing (POCT) platform, test strips have been widely used to analyze OPs along with visible colorimetric strategies, which just supplied qualitative or semi-quantitative detection (Yan et al., 2017; Yan et al., 2019). Because of the aggregation of sensing materials or uneven distribution of recognition units, test strips were lacking sufficient stability and accuracy in real samples analysis (Mahato et al., 2017). To meet the important demands of on-site quantifying OPs, there is a requirement for sensors equipped with solid-phase carriers with convenient and effective recognition. Nanomaterial-based hydrogels are becoming promising materials in the field of sensing and biomedical applications (Yougbare et al., 2020; Patil et al., 2021; Wang H. et al., 2021; Xie et al., 2021; Patel et al., 2022). Taking the advantages of a stable 3D network and friendly environmental protection, sodium alginate (SA) hydrogel has drawn great attention for the encapsulation and immobilization of applications in sensors. As a water-holding network, hydrogels could provide nanometer-scale porous structures for small molecules passing through and trap nanoparticles inside as physical encapsulation, which enhanced the accuracy and stability of the sensors (Campea et al., 2021; Ping et al., 2021; Xu J. et al., 2021; Zhang et al., 2021). Thus, the hydrogel was thought to be a prospective carrier for fabricating the POCT platform. On account of the widespread global coverage of smartphones, the POCT platform assembled with smartphones for tracing pesticides displayed huge potentiality in on-site applications (Jin et al., 2021). Furthermore, 3D-printed technology received extensive attention for its lower cost and its ability to easily produce any shape of smartphone-suited attachments (Wang J. et al., 2021).

Fluorescent nanomaterials including carbon dots (CDs), quantum dots, and silicon dots have been widely used in developing pesticide biosensors, owing to their sensitive responses and particular photoluminescent performance (Aragay et al., 2012; Bartelmess et al., 2015; Xu et al., 2016; Zhi et al., 2019; Lou-Franco et al., 2020; Li et al., 2021c; Su et al., 2021; Yang et al., 2021). For instance, by effectively integrating the optical property of nanomaterials with the catalytic performance of enzymes, the CD-based fluorometric biosensor was successfully developed for the sensitive analysis of pesticides (Wu et al., 2017; Lv et al., 2019). Our group fabricated a fluorometric pesticide sensor using copper nanoclusters with green-emissive fluorescent, possessing better distinction ability than colorimetric sensors (Kong et al., 2019). Although the blue- or green-emissive response acquired good analysis capability toward pesticides, some background interferences of samples led to false-positive events and unsatisfied repeatability. Red-emissive AuNCs with good biocompatibility and splendid anti-interference capability have emerged as versatile indicators (Zhang et al., 2019). Benefiting from a facile one-step green synthesis and structure-controlled property, AuNCs have been utilized to fabricate chem/biosensors for the monitoring of biomarkers and ions. In this study, glutathione-capped AuNCs are selected to construct biosensors for sensing pesticides.

**SCHEME 1:**
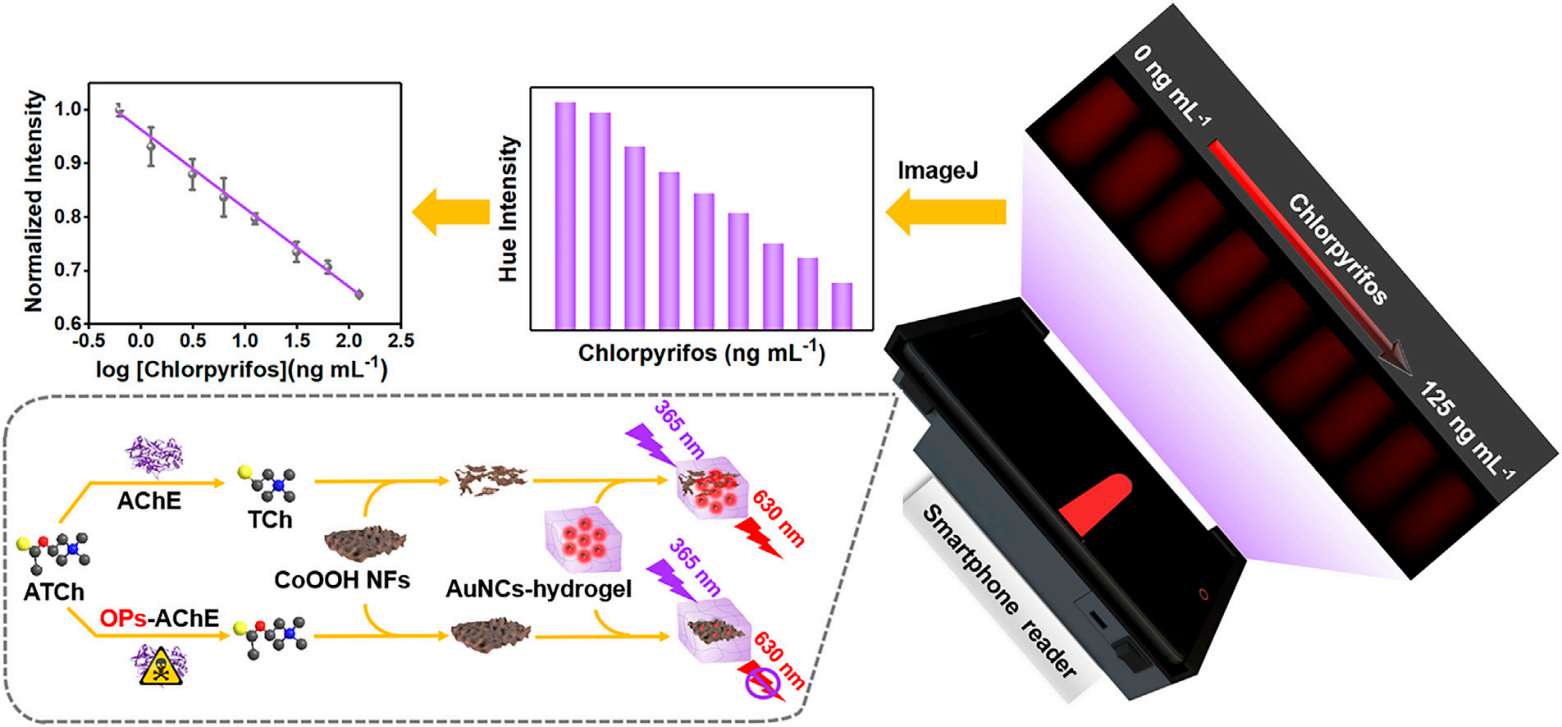
Schematic illustration of the portable fluorescent hydrogel test kit for the on-site quantitation of chlorpyrifos.

Enlightened by the aforementioned work, we fabricated a handheld POCT platform assembled with smartphones for the on-site detection of OPs, possessing satisfactory signal amplification properties. As shown in Scheme 1, the fluorescent hydrogel was acquired by encapsulating the AuNC indicator into the SA hydrogel. By introducing CoOOH NFs, the fluorescence of AuNCs could be efficiently quenched *via* the Förster resonance energy transfer (FRET) effect. As a product of acetylcholinesterase (AChE) catalytic reactions, thiocholine (TCh) could decompose CoOOH NFs into Co^2+^, further controlling the quenching efficiency of CoOOH NFs. Chlorpyrifos, a typical OP, severely restrained the catalytic activity of AChE, and actually limited the yield of TCh and the degradation of CoOOH NFs, leading to a fluorescence response of the AuNC-based hydrogel. To precisely quantify chlorpyrifos, 3D printed subsidiary equipment was combined with a smartphone to catch the fluorescence images, and converted them into digital information by commercial software ImageJ. Notably, by integrating the nanocomposite hydrogel with 3D printed subsidiary equipment, chlorpyrifos was successfully detected in lake water, apple juice, and pear juice with satisfactory results.

## Experimental Section

### Materials and Instruments

Sodium hypochlorite (NaClO), hydrogen tetrachloroaurate hydrate (HAuCl_4_·xH_2_O), and sodium hydroxide (NaOH) were bought from Sinopharm Chemical Reagent Co. Ltd. (Shanghai, China). Calcium chloride (CaCl_2_), SA, glutathione (GSH), ATCh, and cobalt (II) chloride (CoCl_2_) were bought from Aladdin Reagent Co. Ltd. (Shanghai, China). AChE was bought from Ryon Biological Technology Co. Ltd. (Shanghai, China). Tris-HCl buffer solution was obtained from Sigma Aldrich Reagent Co. Ltd. (St. Louis, MO, United States). Deionized water (DI water) with good resistance of 18 MΩ was used in this work. The detailed microstructures of the samples were analyzed with a JEM-7500 scanning electron microscope (SEM) (JEOL, Japan) and a JEM-2100 transmission electron microscope (TEM) (JEOL, Japan). The UV-Vis absorbance results are acquired by using a UV-2550 spectrometer (Shimadzu).

### Preparation of CoOOH Nanoflakes

CoOOH NFs were synthesized in accordance with the previous work (Su et al., 2021): 1 ml of CoCl_2_ (10 mmol L^−1^) solution and 0.3 ml of NaOH (1.0 mol L^−1^) were mixed together first. The mixture was sonicated for 1 min. Then, 50 μl of NaClO (0.9 mol L^−1^) was introduced into the mixed solution and sonicated for 10 min. After that, 650 μl of deionized water was introduced. The CoOOH NFs were collected after centrifuging for 10 min at 10,000 rpm. Then, the products were washed three times with DI water.

### Preparation of AuNC Hydrogel

AuNCs were prepared on the basis of the previously reported approach (Luo et al., 2012). The aqueous solutions of GSH (0.3 ml, 100 mmol/L) and 8.7 ml DI water (resistivity >18 MΩ cm) were successively added into HAuCl_4_ (1.0 ml, 20 mmol/L). The mixture solutions were stirred for 5 min at 25°C. After that, the temperature was heated to 70°C and maintained for 24 h. The red-emission Au clusters were acquired. Then, the AuNC solutions were purified using a 1,000 Da dialysis bag for 24 h. The obtained AuNCs were placed at 4°C.

In a 2ml centrifuge tube, 1.565 ml of the SA (3.83 mg ml^−1^) and 40 μl of AuNCs were mixed. When adding CaCl_2_ (20 μl, 12.5 mg L^−1^) to the aforementioned solution, the hydrogels were generated immediately.

### Hydrogel Test Kit for Chlorpyrifos Sensing

Different concentrations of chlorpyrifos (25 µl) and 25 µl of AChE (0.5 U ml^−1^) were mixed at 37°C for 20 min. Then, 100 µl Tris-HCl (pH 8.0, 10 mmol L^−1^) and 50 µl ATCh (2 mmol L^−1^) were added and reacted at 37°C for 25 min. Subsequently, 75 µl of CoOOH NFs (0.25 mg ml^−1^) and 100 μl deionized water were added at room temperature for 10 min. After that, the mixed solution was added to the prepared AuNC-based hydrogel and equilibrated for 15 s. Finally, the cuvette was interposed into a portable device to catch the fluorescence image under the excitation of a 365-nm laser. The image of the hydrogel was analyzed by ImageJ software.

### Real Sample Detection

Real samples such as lake water, apple juice, and pear juice were prepared as analysis samples to confirm the practical applications of the fluorescent hydrogel sensing platform. The lake water sample (100 ml) was directly used. Apple juice and pear juice were obtained from apples and pears by using a juicer. The supernatant of the apple juice and pear juice was collected after being centrifuged twice at 10,000 rpm for 10 min. Then, the obtained supernatant was diluted 100-fold to measure the chlorpyrifos according to the mentioned process. For the recovery study, certain amounts of chlorpyrifos standard (0.625, 1.25, and 12.5 ng ml^−1^) were spiked into the samples and then evaluated by the described procedure.

## Results and Discussion

### Characterization of AuNCs and CoOOH NFs

**FIGURE 1:**
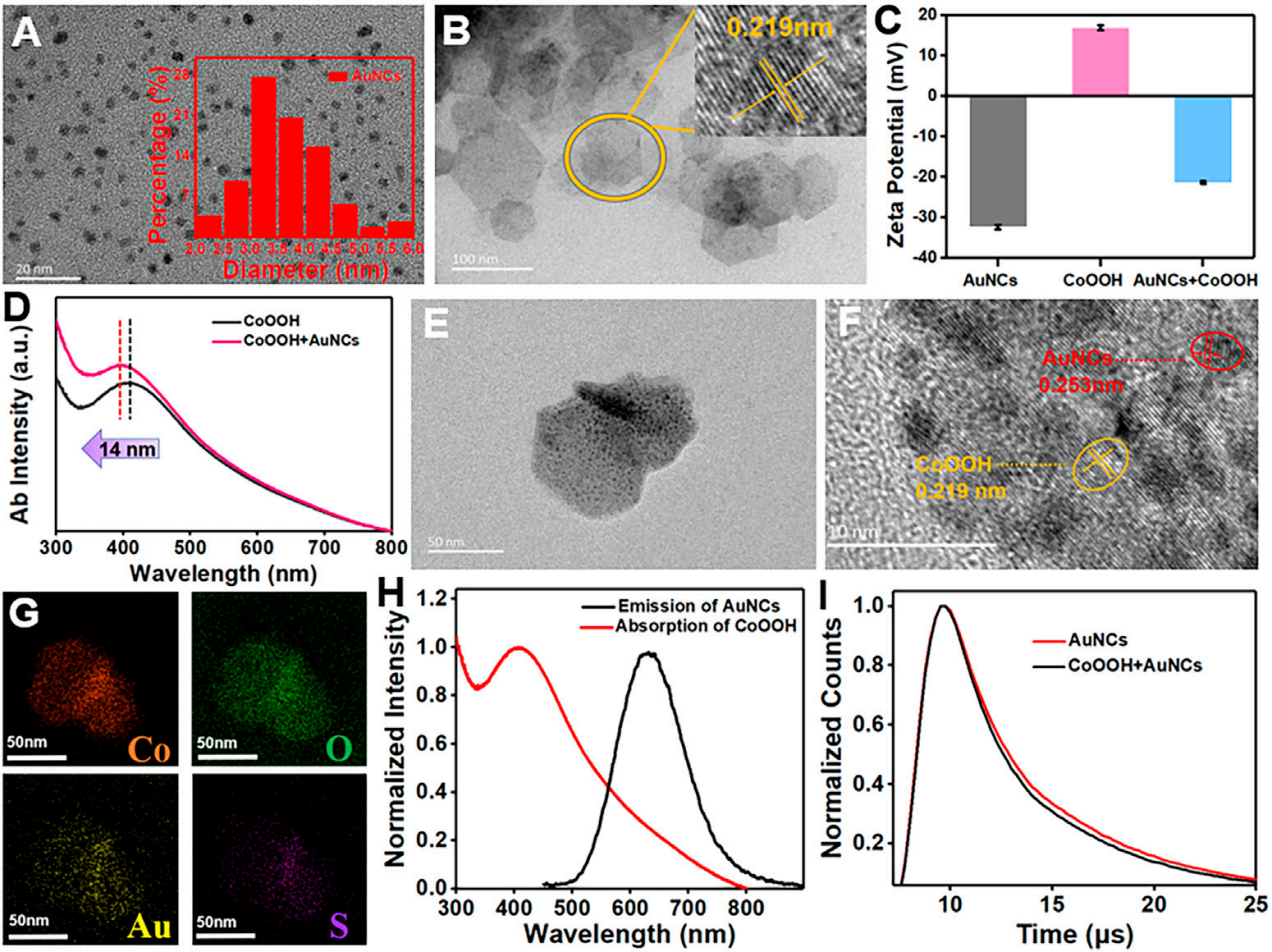
**(A)** TEM image of AuNCs. **(B)** TEM image of CoOOH NFs. **(C)** Zeta potential of CoOOH NFs, AuNCs, and the mixture of CoOOH NFs/AuNCs. **(D)** The UV–vis absorption spectrum of CoOOH NFs and CoOOH NFs + AuNCs. **(E)** TEM image of CoOOH-AuNCs. **(F)** HRTEM image of CoOOH-AuNCs. **(G)** EDS mapping of CoOOH-AuNCs. **(H)** AuNC fluorescence spectrum (black line) and the CoOOH NFs UV–vis absorption spectrum (red line). **(I)** Fluorescence lifetime of AuNCs and AuNCs + CoOOH NFs.

Red-emission AuNCs are simply synthesized by means of a classical reduction approach (Li et al., 2019), using GSH as a reductant and stabilizer. The morphology of AuNCs was characterized using TEM, demonstrating that the mean size is about 3.69 ± 0.75 nm (Figure 1A). The CoOOH NFs as a nanoquencher are prepared by using NaClO as an oxidizer under alkaline conditions, which is composed of CoO_6_ octahedrals and possessed thickness at the nanoscale. As shown in the TEM image, two-dimensional CoOOH NFs possess hexagonal morphology with an average diameter of 78 ± 14.3 nm, which provided a large surface area for loading AuNCs (Figure 1B). The X-ray diﬀraction (XRD) pattern of the obtained CoOOH NFs was consistent with the hexagonal rhomb-centered phase (JCPDS NO. 07-0169), confirming the successful preparation of CoOOH NFs (Supplementary Figure S1). The fabricated CoOOH NFs were also confirmed by Förster transform infrared (FT-IR) spectra. Supplementary Figure S2 shows an absorption peak at 3,300, 1,652, and 540 cm^−1^, relating to the −OH group, double bond (Co-O), and Co-O^2-^ complex vibration of CoOOH NFs, respectively. After introducing the AuNCs (−32.3 mV), the zeta potential of CoOOH NFs (16.8 mV) was changed to be −21.3 mV, forming a forceful electrostatic attraction (Figure 1C). Compared with CoOOH NFs (407 nm), the absorption peak at 393 nm of the CoOOH-AuNCs displayed a 14-nm blue shift, confirming the formation of a CoOOH-AuNC composite (Figure 1D). These results in accordance with the TEM image showed the modification of AuNCs on the CoOOH NF surface (Figures 1E, F). The energy dispersive spectroscopy (EDS) patterns further displayed the elemental composition of Co, O, Au, and S, revealing the combination of CoOOH NFs and AuNCs (Figure 1G). For comparison, the fluorescence emissions of AuNCs (black line) were prominently overlapped by the absorption band of CoOOH NFs (red line) in the range of 500–800 nm (Figure 1H). Furthermore, after adding CoOOH NFs, the fluorescence intensity of AuNCs was obviously quenched (Supplementary Figure S3), and the lifetime of AuNCs changed from 13.31 to 11.3 μs (Figure 1I and Supplementary Table S1). Based on the aforementioned experiments, it is supposed that the quenched principle is ascribed to the FRET effect.

### Sensing Performance Toward Chlorpyrifos

**FIGURE 2:**
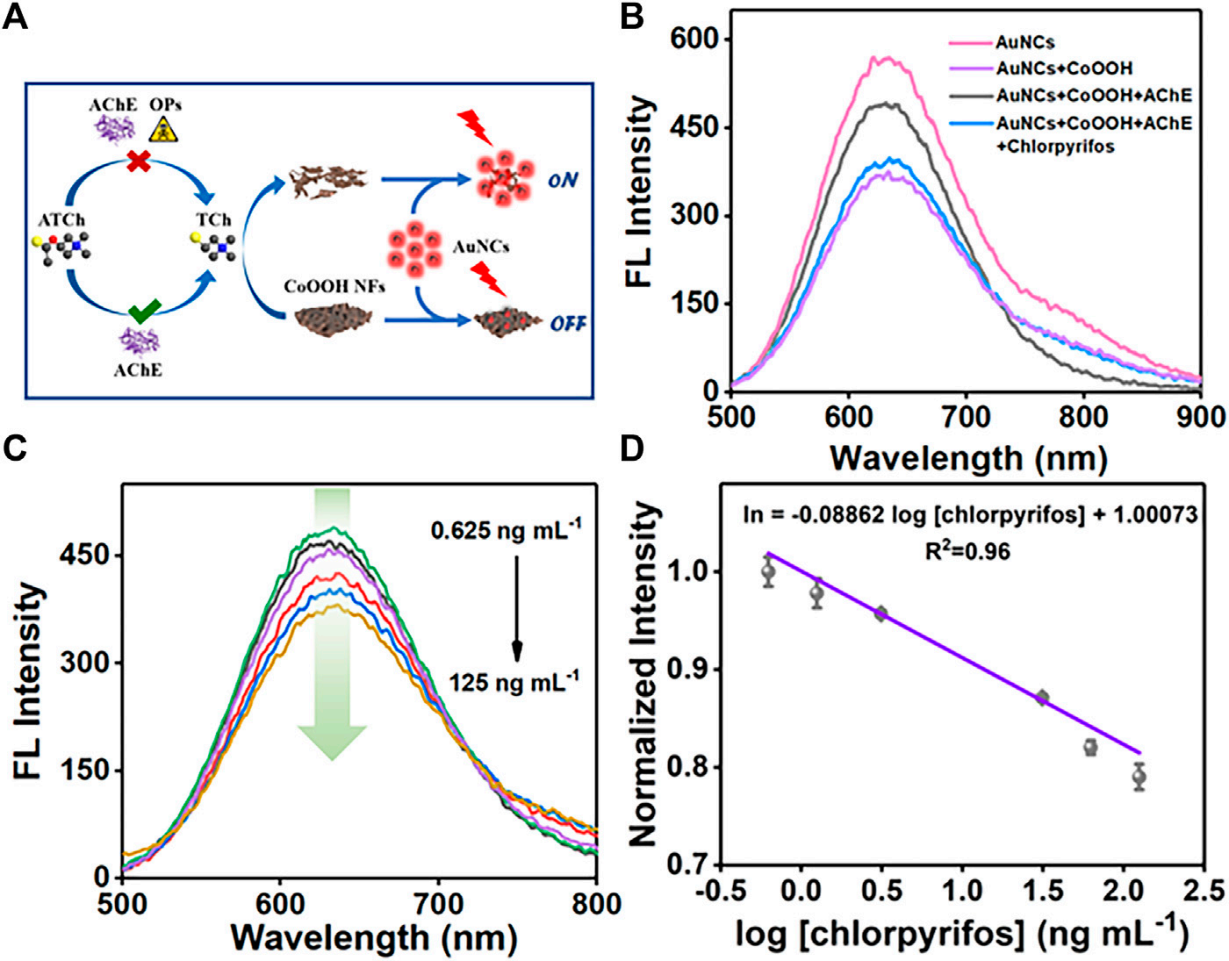
**(A)** Analysis strategy of chlorpyrifos. **(B)** Fluorescence spectra of AuNCs, AuNCs/CoOOH, and AuNCs/CoOOH/AChE/ and AuNCs/CoOOH/AChE + chlorpyrifos. **(C)** Fluorescence intensity of the system toward different concentrations of chlorpyrifos. **(D)** Linear relationship between fluorescence intensity and the logarithm of the concentration of chlorpyrifos.

Combining the fluorescence characteristics of AuNCs with AChE-controlled acetylthiocholine (ATCh) hydrolysis, the fluorometric platform was designed to detect OPs (Figure 2A). The enzyme hydrolysis products are TCh and CH3COOH. The testing system could generate 50 μmol L^−1^ of CH3COOH if ATCh completed catalyzed hydrolysis by AChE. As shown in Supplementary Figure S4, the fluorescence intensity of AuNCs decreased by 2.74% with CH3COOH (50 μmol L^−1^). During the detection process, we add 100 µl Tris-HCl (pH 8.0, 10 mmol L^−1^) buffer to ensure the detection system is in a relatively stable pH environment. TCh could decompose the CoOOH NFs, controlling the quenching efficiency of CoOOH NFs toward AuNCs. Chlorpyrifos inhibits the catalytic activity of AChE, further causing fluorescence response in the system. As shown in Figure 2B, CoOOH NFs could easily quench the fluorescence signal of AuNCs (from the red line to the purple line). When the CoOOH NFs were reduced to Co^2+^ by the enzyme hydrolysis products (TCh), the fluorescence intensity of AuNCs was increased along with the decomposition of CoOOH NFs (black line). Furthermore, the fluorescence intensity of the system could be regulated on account of AChE-induced ATCh hydrolysis (blue line). Owing to the specific inhibition of chlorpyrifos and effective catalyzation toward the substrate, AChE is viewed as an antenna in the sensing system. Therefore, we successfully established the AuNC/CoOOH/AChE/ATCh sensing system to be applied in chlorpyrifos analysis. With the increase of chlorpyrifos, the fluorescence intensity at 630 nm gradually decreased (Figure 2C). As expected, an acceptable linear relationship (R^2^ = 0.96) is obtained with the chlorpyrifos concentrations ranging from 0.625 to 125 ng ml^−1^ (Figure 2D).

### Smartphone Fluorometric Hydrogel Sensing Platform

**FIGURE 3:**
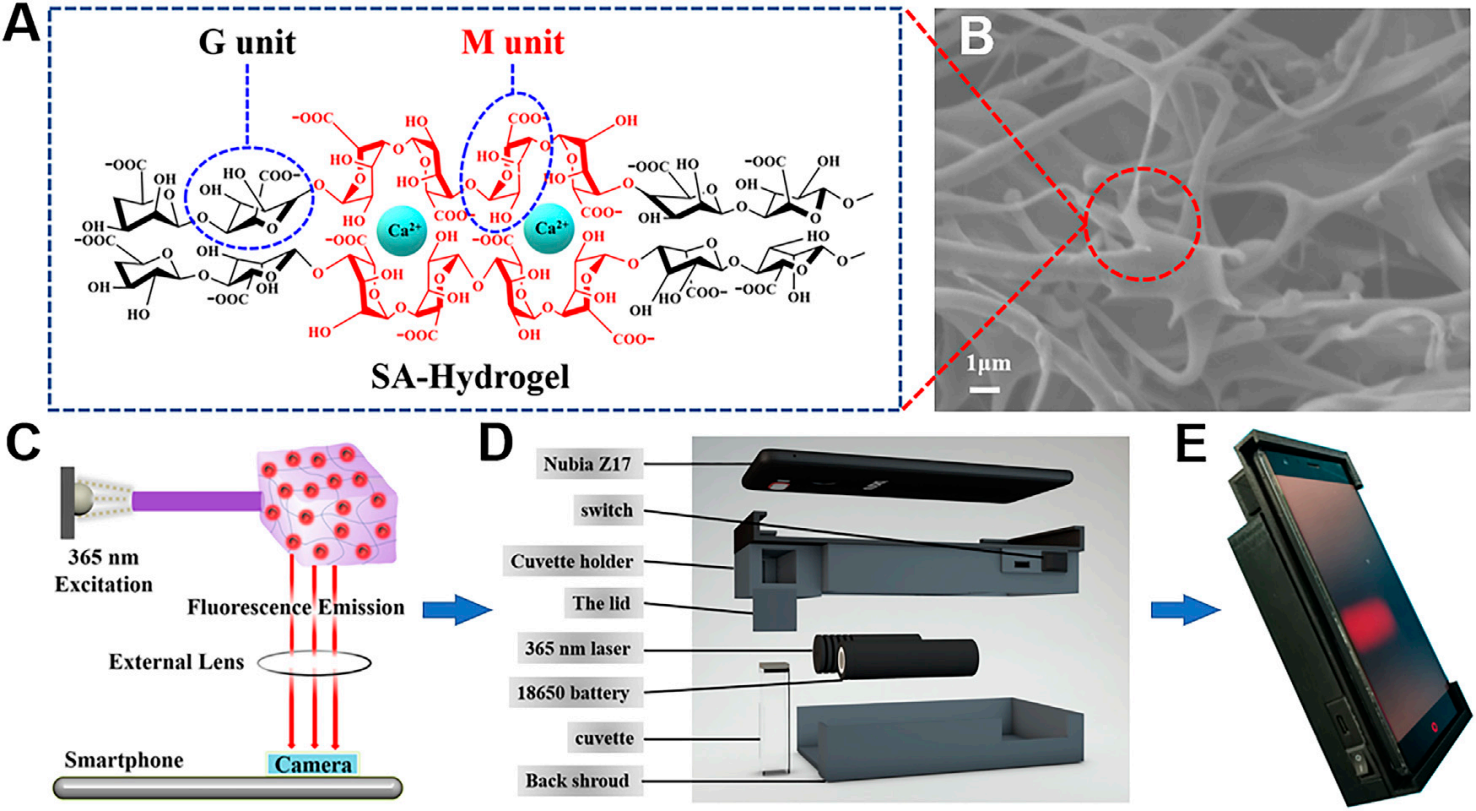
**(A)** Illustration of the “egg-box” structure, containing the G unit and M unit. **(B)** SEM images of SA hydrogels. **(C)** Optical route of the designed portable device. **(D)** Design of the 3D-printed accessory. **(E)** Portable device for image acquisition.

Benefiting from the simple preparation, superb biocompatibility, and 3D networks, hydrogels are selected as a robust carrier in fabricating stimuli-responded POCT platforms. SA, a promising natural polysaccharide, is made up of a β-D-mannuronic acid (M) unit and α-l-guluronic acid (G) unit. After introducing Ca^2+^, the “egg-box” structure is formed because of α-l-guluronic acid (G) unit stacking; thus, the hydrophilic hydrogel is acquired (Figure 3A). The SEM images of the freeze-dried SA hydrogel distinctly demonstrated the layer morphology of porous structures (Figure 3B), possessing a huge capability for nanomaterial anchoring. Considering the aforementioned characteristics, we have easily immobilized AuNCs into the SA hydrogel and constructed a TCh-controlled target response hydrogel test kit for the fluorometric analysis of chlorpyrifos.

**FIGURE 4:**
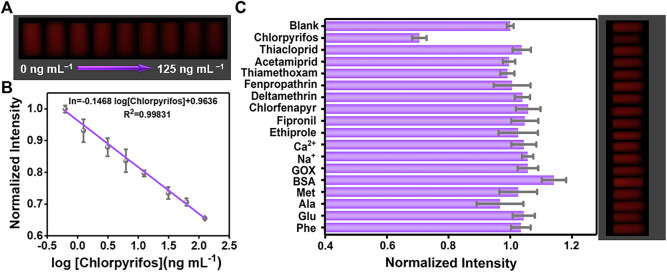
**(A)** Photograph of the test kit with different concentrations of chlorpyrifos from 0 to 125 ng ml^−1^. **(B)** Relationship between normalized intensity and the logarithm of chlorpyrifos concentrations (0.625, 1.25, 3.125, 6.25, 12.5, 31.25, 62.5, and 125 ng ml^−1^). **(C)** Selectivity and the corresponding photograph of the hydrogel kit toward interfering substances.

The conventional detection methods usually consisted of bulky equipment, skilled person, and expensive cost, which could hardly meet the needs of on-site analysis for pesticides. Owing to its excellent capabilities of the digital camera program, good computation, and quick communication, the smartphone has drawn increased attention for developing real-time and portable sample-to-answer detection devices (Celik et al., 2021; Martinez-Avino et al., 2021; Zhu et al., 2021). Furthermore, the 3D printing technology is extensively applied in broad fields because of its low cost and capability to create objects with any predefined shape (Li and Pumera 2021). Hence, we explore a miniaturized handheld detection device that not only takes advantage of the aforementioned superiorities but also immobilizes the relative locations of the light source, sample, and smartphone camera. As shown in Figures 3C–E, the proposed platform is composed of a smartphone, a laser device (6.0 × 2.0 cm) of 365 nm, a four-channel cuvette with 1.0 cm inside diameter, a long-pass filter (1.0 × 1.0 cm), and a 3D-printed accessory (16.0 × 7.8 × 3.8 cm). When catching images, the excitation light is supplied by a 50-mW diode laser. To lower background noise, the long-pass filter with a resisting wavelength at 600 nm is settled between the smartphone camera and the detection sample. Thus, the POCT device is developed by combing the created AuNC/CoOOH/AChE/ATCh target response hydrogel test kit with the miniaturized homemade accessory for quantitatively tracing chlorpyrifos under good specificity and low background interference. Notably, the cost of the handheld POCT device was about $20. Using this handheld detection device, the concentrations of chlorpyrifos could be directly detected by analyzing the captured images (Figure 4A). As expected, an acceptable linear relationship (R^2^ = 0.998) is obtained with chlorpyrifos concentrations ranging from 0.625 to 125 ng ml^−1^ (Figure 4B). The detection limit is calculated to be 0.59 ng ml^−1^ by the equation LOD = (3σ/k), where σ is the standard deviation of blank signals and k is the slope of the calibration curve. It is worth pointing out that the limit of detection (LOD) is 0.59 ng ml^−1^ (S/N = 3), meeting the detection requirements of chlorpyrifos in agricultural products (the maximum residue limit of chlorpyrifos is 20 ng ml^−1^ in fruits). The POCT device provided a low demand for sample concentration (0.625 ng ml^−1^) and a satisfied LOD compared with the previously reported strategies (Supplementary Table S2).

A meaningful index to assess the performance of AuNC-based biosensors is the selectivity toward the target. The common interfering substances such as Na^+^, Ca^2+^, BSA (bovine serum albumin), GOX (glucose oxidase), GSH, Phe (phenylalanine), Met (methionine), Ala (alanine), and other pesticides (pyrazole pesticides, neonicotinoid pesticides, and pyrethroid pesticides) were selected to investigate the selectivity. As shown in Figure 4C, after adding chlorpyrifos or the common interfering substances into the hydrogel system, only chlorpyrifos (62.5 ng ml^−1^) generated a prominent decrease in the fluorescence color. By transforming the color information, a histogram further displayed the normalized intensity of the gray value, exhibiting that the intensity was barely changed by interference substances (125 ng ml^−1^). The good selectivity of the hydrogel test kit for chlorpyrifos detection might ascribe to the excellent specificity of AChE that holds a unique catalyzation effect toward the substrate and specific recognition capability to chlorpyrifos. The outstanding properties for detecting chlorpyrifos might be attributed to the following particular characteristics: 1) The AuNCs as a signal indicator with red-emission could conquer the influence of inevitable autofluorescence of the biological matrix, efficiently blocking background interference and enhancing anti-interference capability. 2) The CoOOH NFs possess high quenching efficiency toward AuNCs on the base of the FRET effect, remarkably improving the detection sensitivity. 3) Compared with the strict pH requirements of acid/alkaline phosphatase and the reversible inhibition of tyrosinase, AChE is the most broadly used enzyme in pesticide detection, which performed specificity recognition toward ATCh and a robust response toward pesticides. 4) SA hydrogels allow small molecules to diffuse through the porous structure and also act as a barrier to avoid the infiltration of biomacromolecules, which are favorable to the stability of the platform.

The practical ability of a hydrogel test kit for pesticide detection is meaningful to environmental assessment and food safety monitoring. To investigate the practical applications of the test kit, we selected lake water, apple juice, and pear juice as test samples. The precision and accuracy of the test kit for chlorpyrifos are investigated using the standard addition method. The recovery rates of chlorpyrifos (0.625, 1.25, and 12.5 ng ml^−1^) were obtained according to the hydrogel test kit. In Supplementary Figure S5, the 25 µl 100-diluted apple juice and pear juice rarely affected the fluorescent signal of AuNCs. As shown in Table 1, the recoveries were determined in the range of 87.47–112.64%, with relative standard derivations (RSDs) lower than 4.43%. Overall, the hydrogel test kit possessed high reliability for monitoring OPs in a complex sample.

**TABLE 1:** Determination results of chlorpyrifos in samples.

Sample	Spiked (ng ml^−1^)	Found (ng ml^−1^)	Recovery (%)	RSD (*n* = 3, %)
Lake water	0.625	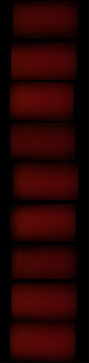 0.67	107.14	4.00
	1.25	1.41	112.64	1.86
	12.5	11.66	93.24	4.43
Apple juice	0.625	0.61	96.95	1.86
	1.25	1.09	87.47	1.71
	12.5	11.13	89.03	3.48
Pear juice	0.625	0.66	105.43	0.29
	1.25	1.22	98.05	1.36
	12.5	11.68	93.42	0.70

## Conclusion

In summary, we demonstrated a fluorescence hydrogel-based POCT platform combined with an AuNC/CoOOH/AChE/TCh system for accurate quantification of chlorpyrifos in a selective and sensitive manner. By using the optical property of AuNCs and the 3D structure of the SA hydrogel, the fluorescence hydrogel with red-emission could reduce the impact of unavoidable self-fluorescence of biotic substrates and the infiltration of biomacromolecules, efficiently improving the anti-interference ability. Furthermore, the CoOOH NFs could efficiently quench the fluorescence of AuNCs based on the FRET effect and quickly recognize AChE-catalyzed hydrolysis products, remarkably improving the detection sensitivity. Notably, a low-cost and straightforward hydrogel test kit has been fabricated by combining the SA hydrogel and 3D-printed accessory, achieving the on-site quantitative analysis of chlorpyrifos with an LOD of 0.59 ng ml^−1^. Such a portable hydrogel test kit has confirmed its accuracy and reliability in real sample detection. Therefore, the prepared hydrogel kit provides a prospective method for the on-site detection of OPs, owning underlying value in food safety management.

## Data Availability

The original contributions presented in the study are included in the article/Supplementary Material, further inquiries can be directed to the corresponding authors.
