# Supplementary material for: A Portable Fluorescent Hydrogel-Based Device for On-Site Quantitation of Organophosphorus Pesticides as Low as the Sub-ppb Level
Source: Front Chem. 2022 Apr 29;10:855281. doi: 10.3389/fchem.2022.855281 (PMC9101059; doi:10.3389/fchem.2022.855281)
Supplement: Supplementary file 1 [file DataSheet1.PDF]

Supplemental Information

A Portable Fluorescent Hydrogel-based Device for On-Site Quantitation of Organophosphorus Pesticide as Low as Sub-ppb Level

Tuhui Wang[a,*], Lening Zhang[a], Hua Xin[a,*]

[a] Department of Thoracic Surgery, China−Japan Union Hospital, Jilin University, Changchun 130033, P. R. China

[*]**Corresponding Author**

Email Address: wangth19@mails.jlu.edu.cn, wangtuhui@qq.com (T. H. Wang)

Email Address: xhua@jlu.edu.cn (H. Xin)

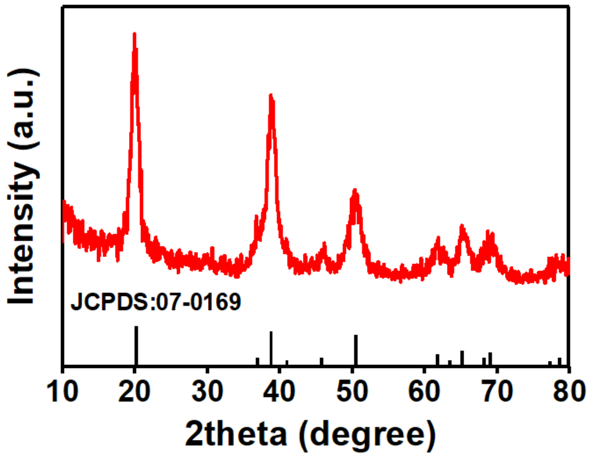

Figure S1. XRD pattern of the CoOOH nanoflakes.

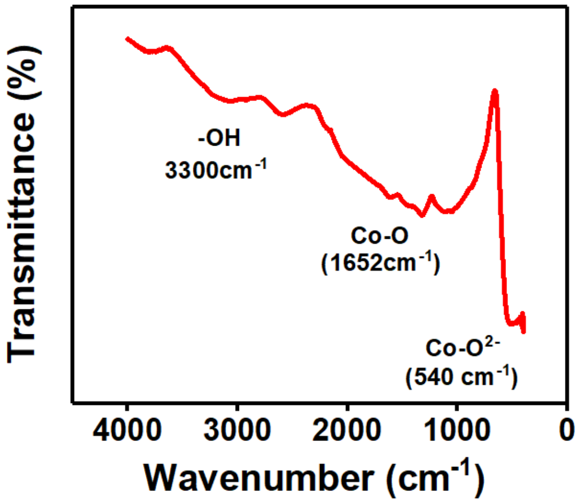

Figure S2. FT-IR spectrum of CoOOH nanoflakes.

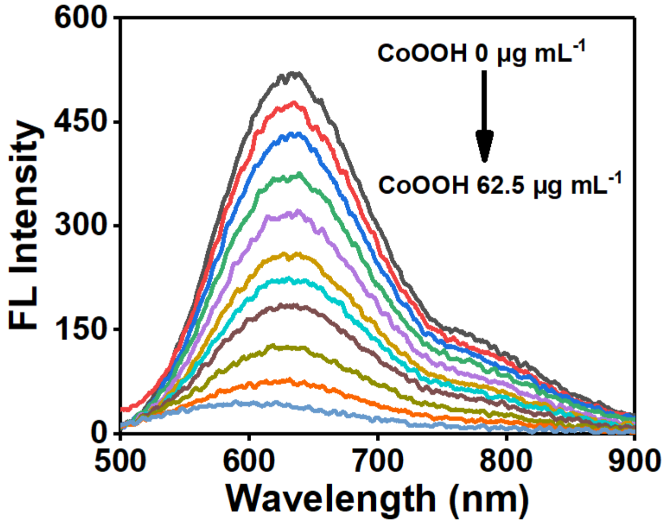

Figure S3. FL intensity of AuNCs toward different concentration of CoOOH nanoflakes (0, 1.25, 3.125, 6.25, 9.375, 12.5, 18.75, 25, 37.5, 50, 62.5 μg mL^{-1}).

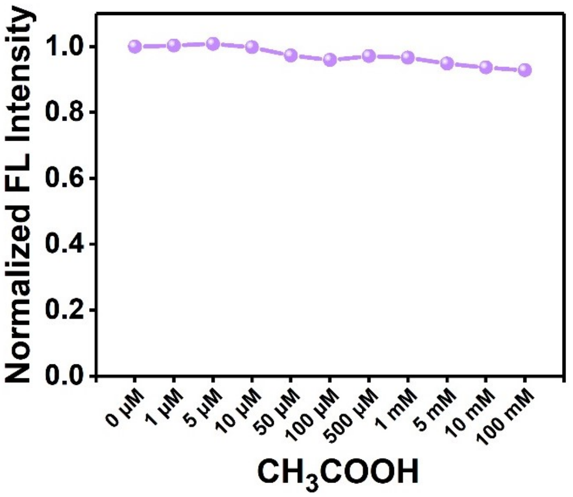

Fig. S4. Fluorescence intensity of AuNCs toward different concentration of CH_3COOH.

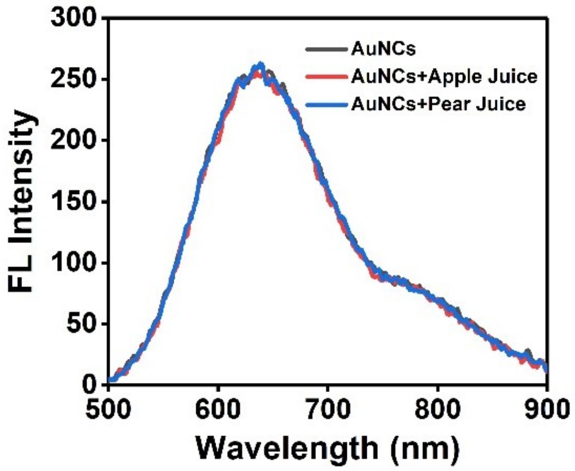

Fig. S5. Fluorescence intensity of AuNCs, AuNCs + apple juice, AuNCs + pear juice.

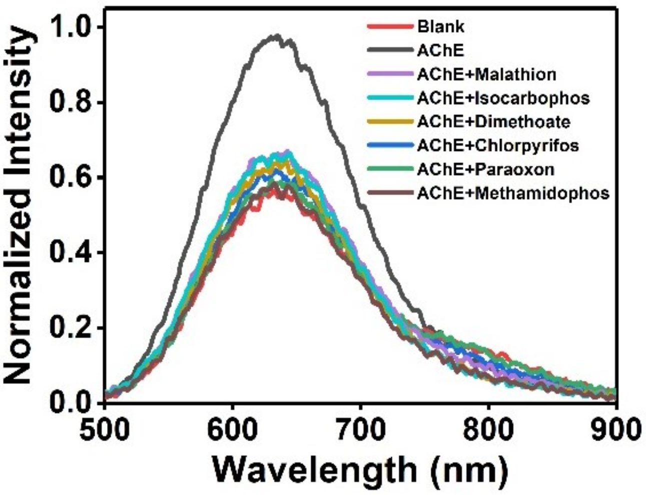

Fig. S6. Fluorescence intensity of system toward different organophosphorus pesticides (125ng mL^{-1}). Calculation of the inhibition efficiency: malathion 76.0%, isocarbophos 78.0%, dimethoate 82.2%, chlorpyrifos 87.8%, paraoxon 89.6%, methamidophos 98.6%.

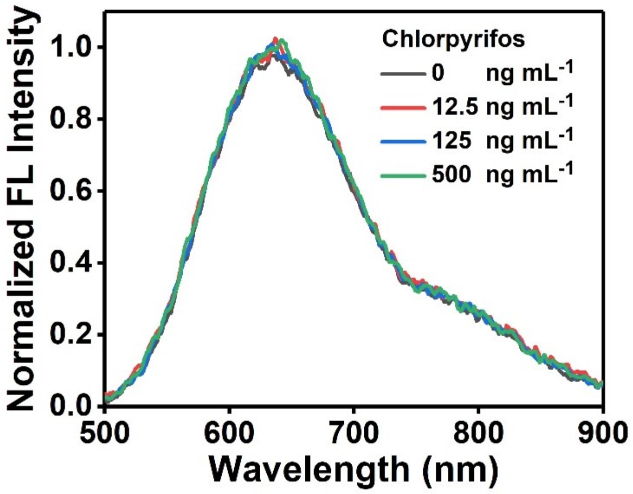

Fig. S7. Fluorescence intensity of AuNCs toward different concentration of chlorpyrifos.

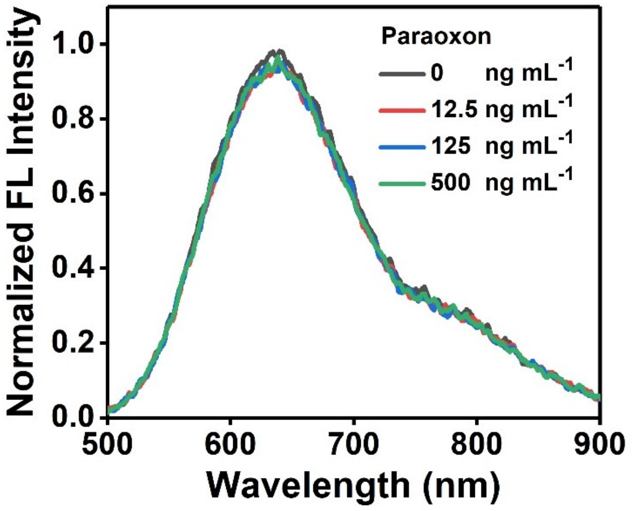

Fig. S8. Fluorescence intensity of AuNCs toward different concentration of paraoxon.

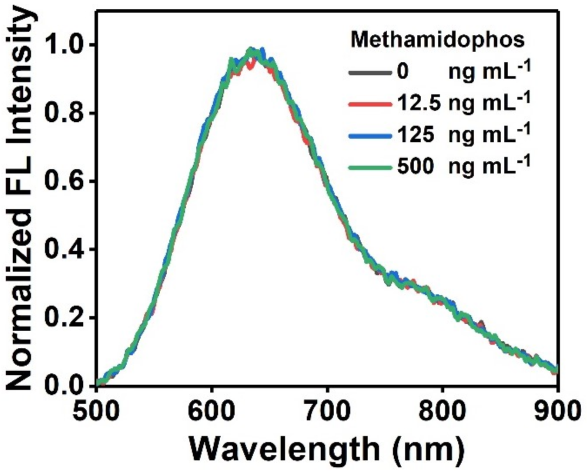

Fig. S9. Fluorescence intensity of AuNCs toward different concentration of methamidophos.

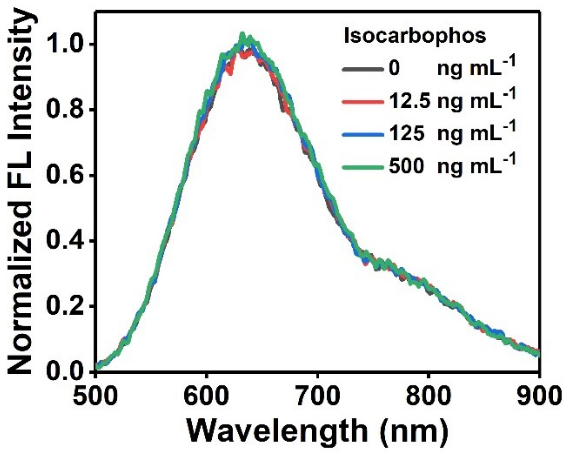

Fig. S10. Fluorescence intensity of AuNCs toward different concentration of isocarbophos.

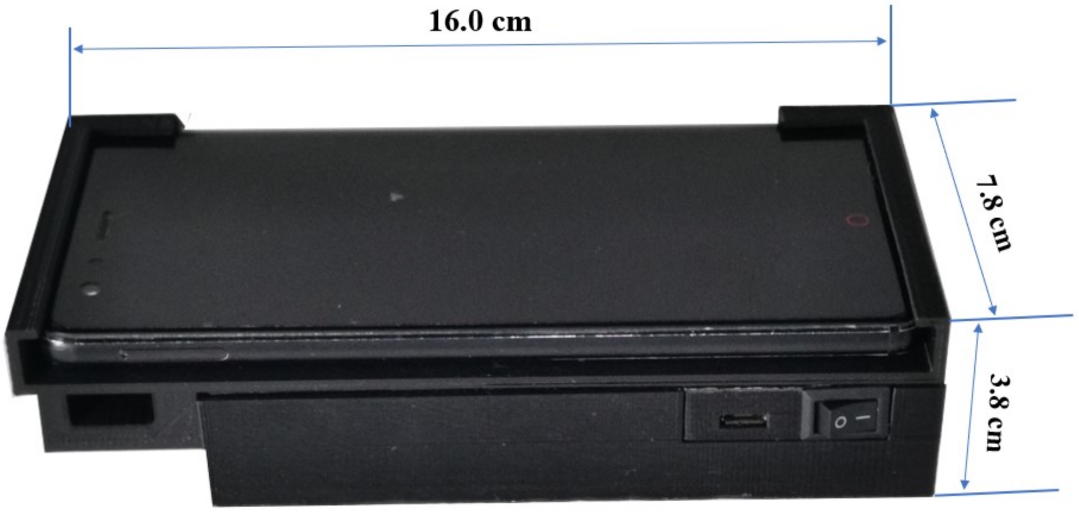

Figure S11. The size illustration of hydrogel portable device.

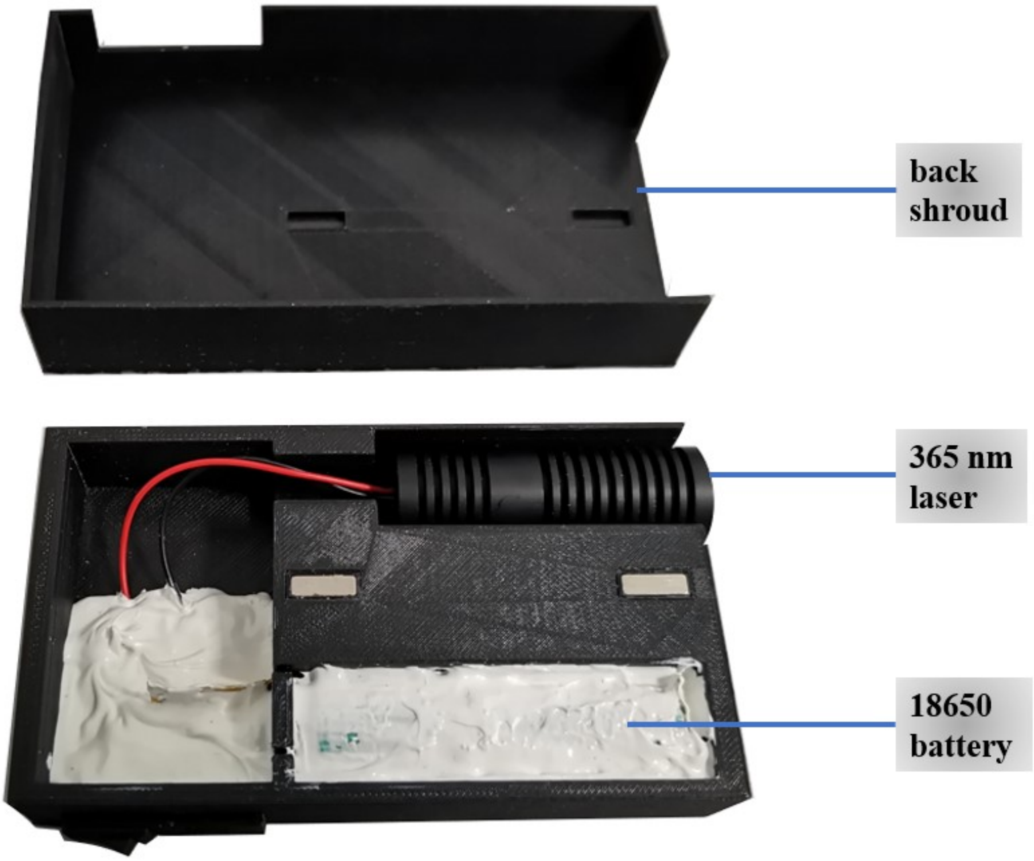

Figure S12. The inner design of portable device.

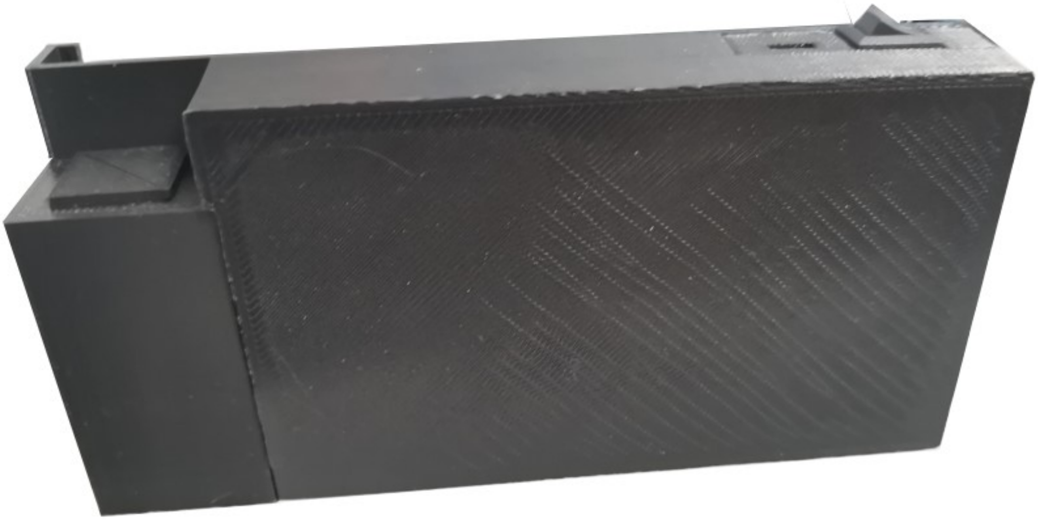

Figure S13. The backside design of hydrogel portable device.

Table S1. The Fit Results of fluorescence lifetime analysis of AuNCs and CoOOH-AuNCs.

Name	τ	Percentage (%)
AuNCs	τ1 (3.080×10^{-6} s)	B1 (36.53)
	τ2 (9.620×10^{-6} s)	B2 (63.25)
	τ3 (1.312×10^{-4} s)	B3 (0.22)
CoOOH-AuNCs	τ1 (2.824×10^{-6} s)	B1 (37.22)
	τ2 (9.219×10^{-6} s)	B2 (62.56)
	τ3 (1.029×10^{-4} s)	B3 (0.22)

Table S2. The analysis performances of different methods for the detection of chlorpyrifos

**Method**	**Linear range ($ng\ mL^{-1}$)**	**Detection limit ($ng\ mL^{-1}$)**	**Reference**
PEC	0.01-80	0.03	(Wang et al. 2021)
Electrochemical aptasensor	0.1 - 150	0.07	(Xu et al. 2018)
Amperometric biosensor	21-122	14	(Guler et al. 2017)
SERS	10-50	10	(Tang et al. 2019)
LC-MS	0.5-100	0.5	(Salm et al. 2009)
Fluorescent aptasensor	1.75-210.35	1.33	(Liu et al. 2019)
Electrochemical aptasensor	$1\text{-}10^5$	0.33	(Jiao et al. 2016)
Electrochemical aptasensor	$0.1\text{-}10^5$	0.033	(Jiao et al. 2017)
Colorimetry	2-20	0.57	(Nana et al. 2021)
Fluorescence Hydrogel test kit	0.625-125	0.59	This work

Reference

Guler, M., Turkoglu, V., Basi, Z. (2017). Determination of malation, methidathion, and chlorpyrifos ethyl pesticides using acetylcholinesterase biosensor based on Nafion/Ag@rGO-NH2 nanocomposites, Electrochim Acta 240, 129-135. doi:10.1016/j.electacta.2017.04.069

Jiao, Y.C., Hou, W.J., Fu, J.Y., Guo, Y.M., Xia, S., Wang, X.Y., et al. (2017). A nanostructured electrochemical aptasensor for highly sensitive detection of chlorpyrifos, Sens. Actuators, B 243, 1164-1170. doi:10.1016/j.snb.2016.12.106

Jiao, Y.C., Jia, H.Y., Guo, Y.M., Zhang, H.Y., Wang, Z.Q., Sun, X., et al. (2016). An ultrasensitive aptasensor for chlorpyrifos based on ordered mesoporous carbon/ferrocene hybrid multiwalled carbon nanotubes, RSC Adv. 6(63), 58541-58548. doi:10.1039/c6ra07735h

Liu, Q.J., Wang, H., Han, P., Feng, X.Y. (2019)., Fluorescent aptasensing of chlorpyrifos based on the assembly of cationic conjugated polymer-aggregated gold nanoparticles and luminescent metal-organic frameworks, Analyst 144(20), 6025-6032. doi:10.1039/c9an00943d

Nana, L., Ruiyi, L., Qinsheng, W., Yongqiang, Y., Xiulan, S., Guangli, W., et al. (2021). Colorimetric detection of chlorpyrifos in peach based on cobalt-graphene nanohybrid with excellent oxidase-like activity and reusability. J. Hazard. Mater. 415, 125752. doi:10.1016/j.jhazmat.2021.125752

Salm, P., Taylor, P.J., Roberts, D., de Silva, J. (2009). Liquid chromatography-tandem mass spectrometry method for the simultaneous quantitative determination of the organophosphorus pesticides dimethoate, fenthion, diazinon and chlorpyrifos in human blood, J. Chromatogr. B 877(5-6), 568-574. doi:10.1016/j.jchromb.2008.12.066

Tang, J.S., Chen, W.W., Ju, H.X. (2019). Rapid detection of pesticide residues using a silver nanoparticles coated glass bead as nonplanar substrate for SERS sensing, Sens. Actuators, B 287, 576-583. doi:10.1016/j.snb.2019.02.084

Wang, H., Liang, D., Xu, Y., Liang, X., Qiu, X., Lin, Z. (2021). A highly efficient photoelectrochemical sensor for detection of chlorpyrifos based on 2D/2D β-Bi2O3/g-C3N4 heterojunctions. Environ. Sci.: Nano 8(3), 773-783. doi:10.1039/d0en01243b

Xu, G.L., Huo, D.Q., Hou, C.J., Zhao, Y.N., Bao, J., Yang, M., et al. (2018). A regenerative and selective electrochemical aptasensor based on copper oxide nanoflowers-single walled carbon nanotubes nanocomposite for chlorpyrifos detection, Talanta 178, 1046-1052. doi:10.1016/j.talanta.2017.08.086
